# Incidental Unilateral Tuberculous Sacroiliitis Detected by ^18^F-FDG PET/CT in a Patient with Abdominal Tuberculosis

**DOI:** 10.22038/aojnmb.2017.8634

**Published:** 2017

**Authors:** Domenico Albano, Giorgio Treglia, Paolo Desenzani, Francesco Bertagna

**Affiliations:** 1 Nuclear Medicine, University of Brescia and Spedali Civili Brescia, Brescia, Italy; 2 Department of Nuclear Medicine and PET/CT Center, Oncology Institute of Southern Switzerland, Bellinzona, Switzerland; 3 Division of Internal Medicine, Montichiari Hospital, Brescia, Italy

**Keywords:** Abdominal tuberculosis, ^18^F-FDG, PET/CT, Sacroiliitis

## Abstract

Tuberculosis is a systemic disease which involves skeletal and articular system very rarely. Osteoarticular tuberculosis commonly occurs in the vertebral column and more rarely in the sacroiliac joints. In this study, we report a 44-years-old male patient with low-grade fever, malabsorption syndrome, abdominal and pelvic ascites and low-back pain, that underwent ^18^F-FDG PET/CT for identifying the cause of signs and symptoms after a negative abdominal CT and negative thorax radiography. The study revealed increased tracer uptake at the peritoneal ascites and at the right sacroiliac joint in absence of bone alteration suggesting a sacroiliitis. Staining of the ascitic fluid was positive for acid-fast bacilli (Ziehl–Neelsen) and in the subsequent abdominal paracentesis Mycobacterium Tuberculosis was isolated; the final diagnosis was abdominal tuberculosis with a sacroiliac joint involvement. The patient started antitubercular therapy for 6 months and the clinical conditions were resolved, in particular both back pain and ascites disappeared.

## Introduction

Sacroiliitis is an inflammation of one or both of sacroiliac joints which appears often with low back pain and with possible extension down to one or both legs. It shows a vague and non specific clinical presentation so it can be difficult to diagnose. Tuberculous infections of musculoskeletal system are very rare (about 1-5% of cases) and the sacroiliac joint is involved in 3-9, 7% (1). Also tuberculous sacroiliitis is difficult to recognize because it may be mistaken for other causes of low back pain like traumatic injuries, spondyloarthropathies, osteoarthritis, bacterial infections, pregnancy or idiopathic (2). Low-back pain is the most common symptom but is less specific. Plain radiographs may not show any abnormality in the early stage of disease; computed tomography (CT) and magnetic resonance imaging (MRI) are more helpful but they have some limits and often there are not diagnostic (2, 3). Bone scan can be useful for early recognition but it has low sensitivity (4). Fluorine-18-fluorodeoxyglucose positron emission tomography/computed tomography (^18^F-FDG PET/CT) is a metabolic imaging technique that has been used for diagnosing cancers of various organs, but has been demonstrated to be helpful also in the field of infection and inflammation, especially to determine the extension and severity of disease, like in tuberculosis (5). In our case ^18^F-FDG PET/CT was helpful to detect diffuse uptake at the peritoneal ascites and at right sacroiliac joint, leading to the diagnosis of abdominal tuberculosis with unilateral sacroiliitis and starting an adequate treatment.

## Case report

**Figure 1:**
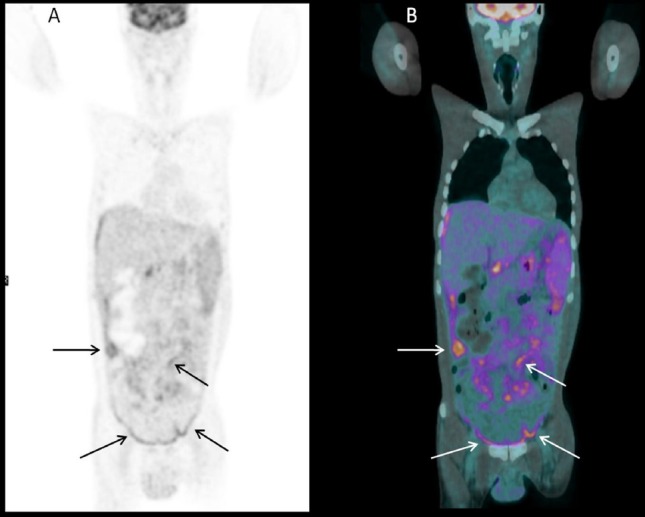
Coronal PET (A) and fused PET/CT (B) images showing high FDG uptake at the abdominal and pelvic peritoneal cavity. Black and white arrows indicate the most intense sites of tracer uptake.

**Figure 2:**
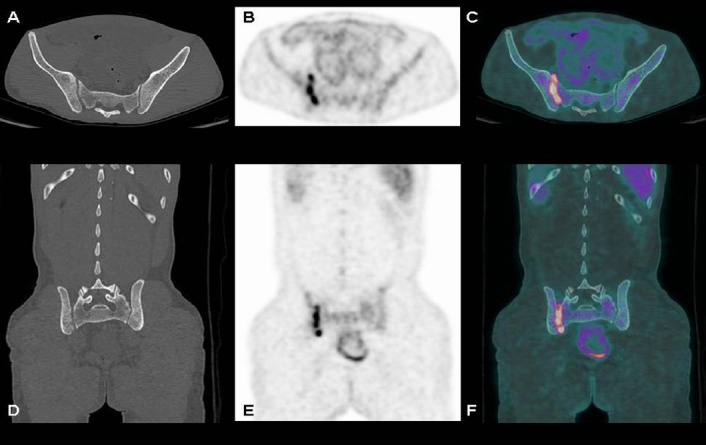
Anterior axial CT (A), PET (B), and fused PET/CT (C) images, coronal CT (D), PET (D) and fused PET/CT (E) images documenting increased tracer uptake at the right sacroiliac joint (SUV_max_=8.9) in absence of bone alteration, suggesting the presence of a sacroiliitis.

A 44-years-old male patient with low-grade fever, malabsorption syndrome, diffuse abdominal and pelvic ascites, low-back pain, erythrocyte sedimentation rate and C-reactive protein elevation underwent ^18^F-FDG PET/CT for identifying the cause of these signs and symptoms after a negative abdominal CT and negative thorax radiography. PET/CT was acquired 60 minutes after the intravenous injection of 3.5 MBq/Kg of ^18^F-FDG on Discovery 690 tomograph (GE-Milwaukee, Wi, USA; 64-slice-CT, 80 mA, 120 Kv; 2.5 minutes/bed; 256×256 matrix, 60 cm field of view). The glucose level was 84 mg/dl. ^18^F-FDG-PET/CT showed high uptake of ^18^F-FDG at the entire peritoneal cavity (Figure 1) and at some mediastinal (one subcarinal and two internal mammary) and abdominal (one superior diaphragmatic, one interaortocaval and one hepatic hilum) nodes. Moreover PET/CT revealed high tracer uptake at right sacroiliac joint with a standardized uptake value (SUV) max=8.9 (Figure 2). Staining of the ascetic fluid was positive for acid-fast bacilli (Ziehl–Neelsen) and in the subsequent abdominal paracentesis Mycobacterium Tuberculosis was isolated, diagnosing an abdominal tuberculosis with a unilateral sacroiliac joint involvement. So the patient started anti-tubercular therapy with isoniazid, rifampin, pyrazinamide and ethambutol for 6 months. After a few weeks the symptoms disappeared and an abdominal CT and MRI showed no peritoneal ascites. Also the buttock pain disappeared. At the end of the therapy, a PET/CT was made and showed no uptake in peritoneal cavity and sacroiliac joints.

## Discussion

Tuberculosis is a systemic disease which has extrapulmonary involvement in 15-20% of cases; joint involvement is very rare (6). Gastrointestinal tuberculosis is the sixth most frequent form of an extrapulmonary site, after lymphatic, genitourinary, bone and joint, miliary and meningeal tuberculosis (7). Sacroiliitis is commonly diagnosed and evaluated by radiograph, CT and MRI which are valuable methods for defining the extent of involvement and complications but often inadequate to detect early disease, as patients may have symptoms for several years before morphological abnormalities can be seen (8, 9). Despite radiological imaging as radiography, CT and MRI are the first approach in this disease and are useful in evaluating sacroiliitis, as demonstrated by van Gaalena et al. (8), no progress has been made in radiography of the sacroiliac joints in recent years. In particular, the number of high-quality studies on the diagnostic utility of MRI of the sacroiliac joints remains relatively limited. Nuclear medicine techniques (including three-phase technetium-99m-methylene-diphosphonate bone scintigraphy and PET/CT with different tracers) can diagnose a sacroiliitis earlier than morphological imaging. In particular the role of PET/CT with different tracers in inflammatory diseases is continuously growing (10). To date, very few cases of sacroiliitis detected or evaluated by ^18^F-FDG PET/CT (11-13) or ^18^F-fluoride-PET/CT (14, 15) are currently available in literature. Only one case of tuberculous sacroiliitis detecting by PET/CT is described by Ozmen et al. in a patient with multisystemic tuberculosis involving pleura, column, mediastinal and abdominal nodes, and sacroiliac joint (16).

In this situation metabolic imaging can favourably fit usefully in detecting inflammatory phenomenon in an earlier stage of disease. ^18^F-FDG is a glucose analogue which is taken up both by neoplastic and inflammatory cells like neutrophils, lymphocytes and macrophages because all these cells have increased glucose metabolism and use glucose as a source of energy. Furthermore, as whole-body imaging, ^18^F-FDG PET/CT is able to detect other inflammatory lesions throughout the body which can be associated to sacroiliitis (17).

In our case, the unexpected unilateral sacroiliac joint uptake of ^18^F-FDG allowed an early diagnosis of sacroiliitis, excluding other possible causes of the symptoms in a patient with abdominal tuberculosis. Detection of FDG uptake, although there were no pathological findings on radiograph and CT images of the right sacroiliac joint, supports the idea that FDG PET may be more sensitive, especially at early stages of inflammation before the occurrence of anatomical changes.

In this case, a complete symptomatic response was obtained at all involved parts of the body after a few weeks of tuberculostatic treatment. This indicates that PET/CT was important to detect abdominal tubercoulosis with sacroiliac joint involvement, guiding the management of the patient, in particular the therapy and evaluating the remission.
